# Real‐World Outcomes of Polatuzumab Vedotin Plus R‐CHP Versus R‐CHOP‐Based Regimens in Japanese Patients With Untreated Diffuse Large B‐Cell Lymphoma

**DOI:** 10.1002/cam4.71531

**Published:** 2026-01-12

**Authors:** Masaaki Hotta, Atsushi Satake, Ayako Iwama, Tokiko Hoshiyama, Yukie Tsubokura, Hideaki Yoshimura, Shinya Fujita, Yumiko Kono, Tomoki Ito

**Affiliations:** ^1^ First Department of Internal Medicine Kansai Medical University Osaka Japan; ^2^ Department of Radiology Kansai Medical University Osaka Japan

**Keywords:** diffuse large B‐cell lymphoma, polatuzumab vedotin, PV‐R‐CHP, R‐CHOP

## Abstract

**Background:**

PV‐R‐CHP (polatuzumab vedotin, rituximab, cyclophosphamide, doxorubicin, and prednisone) is a recently introduced regimen for untreated diffuse large B‐cell lymphoma (DLBCL). However, real‐world data comparing its efficacy and safety with those of R‐CHOP (rituximab, cyclophosphamide, doxorubicin, vincristine, and prednisone) remain limited.

**Aims:**

This study aimed to evaluate the real‐world efficacy and safety of PV‐R‐CHP compared with R‐CHOP‐based regimens in patients with newly diagnosed DLBCL.

**Materials and Methods:**

We conducted a retrospective analysis of patients with DLBCL who received PV‐R‐CHP or R‐CHOP‐based treatment as first‐line therapy at Kansai Medical University Hospital between January 2020 and August 2023. The primary endpoint was progression‐free survival (PFS). Other outcomes included overall survival (OS), treatment response, subgroup analyses, and adverse events. Propensity score matching was performed, and prespecified subgroup analyses were conducted in the matched cohort.

**Results:**

A total of 153 patients were included: 53 received PV‐R‐CHP and 100 received R‐CHOP. At 1 year, both PFS and OS were significantly higher in the PV‐R‐CHP group than in the R‐CHOP group (PFS: 89.3% vs. 70.9%, hazard ratio [HR] 0.30 [95% confidence interval (CI): 0.12–0.78], *p* = 0.013; OS: 92.5% vs. 80.0%, HR 0.28 [95% CI: 0.08–0.95], *p* = 0.041). Complete response rates were comparable between the groups (81.1% vs. 76.0%, *p* = 0.543). In propensity score–matched analyses, PV‐R‐CHP was associated with improved PFS in patients with non–germinal center B‐cell–like (non‐GCB) DLBCL. The incidence of grade ≥ 3 adverse events was similar between the regimens, with lymphopenia being the most frequent toxicity.

**Conclusion:**

PV‐R‐CHP may offer improved survival outcomes compared with R‐CHOP in newly diagnosed DLBCL, particularly in patients with non‐GCB, with an acceptable safety profile in a real‐world setting. Although longer follow‐up is required to confirm durability, these findings support the use of PV‐R‐CHP as a frontline treatment option.

## Graphical abstract

PV‐R‐CHP showed improved progression‐free and overall survivals compared to R‐CHOP‐based regimens in newly diagnosed Japanese patients with DLBCL, particularly in those with non‐GCB subtype. These results were consistent in both the overall cohort and propensity score‐matched cohorts.

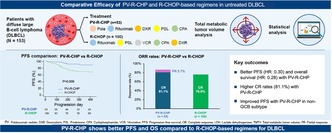

## 1 Introduction

Diffuse large B‐cell lymphoma (DLBCL) constitutes the most prevalent and aggressive subtype of non‐Hodgkin lymphoma, representing over 30% of such cases in adults globally [1, 2, 3]. CHOP (cyclophosphamide [CPA], vincristine [VCR], doxorubicin [DXR], and prednisolone) combined with rituximab (R‐CHOP), the first anti‐CD20 monoclonal antibody, is the standard treatment for untreated DLBCL [4, 5, 6]. Approximately two‐thirds of patients newly diagnosed with DLBCL are cured when treated with R‐CHOP; however, the rest have refractory or relapsed disease [7]. The development of other, more effective regimens has been constrained. Several approaches have been taken in randomized trials to improve R‐CHOP treatment outcome; however, dose‐dense strategies [8, 9, 10, 11], second‐generation anti‐CD20 monoclonal antibodies [12], or novel agents [13, 14] have not succeeded.

Polatuzumab vedotin (PV) is an antibody–drug conjugate with an anti‐CD79b monoclonal antibody conjugated through a protease‐cleavable linker to monomethyl auristatin E, a potent microtubule inhibitor. PV combined with rituximab and bendamustine demonstrates reduced risk of death and manageable toxicity in transplantation‐ineligible patients with relapsed/refractory DLBCL compared with rituximab and bendamustine [15]. Alternatively, PV combined with rituximab, CPA, DXR, and prednisone (PV‐R‐CHP) showed a significantly prolonged progression‐free survival (PFS) compared to R‐CHOP (hazard ratio [HR] 0.73 [95% confidence interval (CI): 0.57–0.95]; *p* = 0.02, 2‐year PFS: 76.7% vs. 70.2%) in the POLARIX study, an international, placebo‐controlled phase 3 trial, on patients with previously untreated DLBCL [16]. Therefore, PV‐R‐CHP was approved in Japan for previously untreated DLBCL in August 2022. PV‐R‐CHP and R‐CHOP are the current standard treatments for previously untreated DLBCL. However, the real‐world efficacy and safety of PV‐R‐CHP compared to R‐CHOP remain unclear. After PV became commercially available, we implemented PV‐R‐CHP as the standard treatment for untreated DLBCL at our institution. This study retrospectively compared the efficacy and toxicity of PV‐R‐CHP to those of R‐CHOP at our institution to clarify the real‐world impact of PV‐R‐CHP in a Japanese patient setting.

## 2 Methods

### 2.1 Patient Population

Consecutive patients diagnosed with *de novo* DLBCL, not otherwise specified, or DLBCL transformed from indolent lymphomas and treated with PV‐R‐CHP, R‐CHOP, or rituximab, pirarubicin, CPA, VCR, and prednisolone (R‐THP‐COP) between January 2020 and August 2023 at Kansai Medical University Hospital were included in this study. In our institution, R‐THP‐COP was used as an alternative to R‐CHOP in patients aged ≥ 80 years or those with a history of cardiac diseases, based on previous reports suggesting that THP‐COP has a comparable or superior cardiotoxicity profile to that of CHOP while maintaining similar efficacy in older adult patients with B‐cell lymphomas [17]. All patients treated after August 2022 received PV‐R‐CHP. This study included treatment‐naïve DLBCL patients aged ≥ 18 years who were administered at least 1 cycle of PV‐R‐CHP, R‐CHOP, or R‐THP‐COP and had complete clinical and laboratory data available. Cell of origin (COO) was determined using the Hans algorithm by immunochemistry [18]. The International Prognostic Index (IPI) was calculated based on the standard definition [19]. Bulky lesions were defined as lesions with a maximum diameter ≥ 7.5 cm. All research procedures conformed to the Declaration of Helsinki and were sanctioned by the institutional review board of the Faculty of Medicine, Kansai Medical University. Participants provided written informed consent through an opt‐out approach as detailed on the institution's website.

### 2.2 Treatment and Calculation of Relative Dose Intensity

PV‐R‐CHP consisted of intravenous PV (1.8 mg/kg on day 1), intravenous rituximab (375 mg/m^2^ on day 1), and CHP (intravenous CPA 750 mg/m^2^ on day 1; intravenous DXR 50 mg/m^2^ on day 1; intravenous or oral PSL 100 mg/body on days 1–5) [16]. R‐CHOP consisted of intravenous rituximab (375 mg/m^2^ on day 1) and CHOP (intravenous CPA 750 mg/m^2^ on day 1; intravenous DXR 50 mg/m^2^ on day 1; intravenous VCR 1.4 mg/m^2^ [maximum 2 mg/body] on day 1; intravenous or oral PSL 100 mg/body on days 1–5). THP‐COP used pirarubicin 30 mg/m^2^ instead of DXR. As THP‐COP itself is a regimen developed for older adult patients, in which the pirarubicin dose (30 mg/m^2^) was considered approximately equivalent to 30 mg/m^2^ of DXR in efficacy, the regimen also involves the reduction of other agents (CPA, VCR, and prednisolone), resulting in lower overall toxicity compared with 2/3‐dose CHOP. Therefore, additional dose reductions were not commonly prescribed. After PV‐R‐CHP was approved, PV‐R‐miniCHP was increasingly adopted in older adult patients who had previously been treated with R‐THP‐COP. In contrast to the POLARIX study, two additional doses of rituximab were not routinely administered after 6 cycles of therapy in either the PV‐R‐CHP or R‐CHOP groups in this study. In addition to systemic chemotherapy, prophylactic intrathecal chemotherapy (three or four 15 mg doses of methotrexate, between cycles 1 and 6) was administered to some patients at the treating physician's discretion, based on central nervous system (CNS)‐IPI risk stratification and site‐specific high‐risk involvement [20]. Every 3 weeks, the treatment course was repeated and the physician adjusted the dosage according to their clinical judgment. Dose intensity was calculated with the following formula: planned dose per course (mg/m^2^)/planned period per day. To calculate the relative dose intensity (RDI) (%), dose intensity was divided by the respective target dose intensity and multiplied by 100. The protocol‐planned doses for standard R‐CHOP were fixed as the target for patients treated with R‐THP‐COP. The average RDI (ARDI) was the average actual RDI of each chemotherapeutic drug (PV‐R‐CHP, PV, rituximab, DXR, and CPA; R‐CHOP/THP‐COP, rituximab, DXR or THP, CPA, and VCR) of each cycle [21]. The total ARDI (tARDI) was the average ARDI of each cycle during the entire treatment period.

### 2.3 Tumor Volume Analysis

Baseline total metabolic tumor volume (TMTV) analyses were performed using a gradient method [22] with the threshold value set using AW Server software (version 3.2, GE Healthcare). For non‐FDG avid cases where MTV was visually overestimated using the gradient method, the radiologist individually reestablished the threshold. TMTVs were calculated from the sum of all MTVs of individual lesions. Patients with no PET‐CT‐evaluable lesions were excluded from the TMTV analyses.

### 2.4 Assessments and Definitions

PFS was chosen as the primary endpoint, reflecting the duration between therapy initiation and either relapse, progression, or death—whichever occurred first. Secondary endpoints were overall survival (OS) (time from treatment initiation to death of any cause), response rate at the end of treatment, PFS/OS in subgroups (COO, IPI, Revised IPI [R‐IPI]), and treatment‐related adverse events (AEs). R‐IPI scores were calculated as originally described [23]. Positron emission tomography‐computed tomography (PET‐CT) scans were conducted after the last cycle to assess response according to the Lugano classification [24] for patients who completed treatment. Patients without a PET‐CT‐evaluable lesion at baseline were excluded from the response rate calculation but included in the PFS and OS analyses. Patients who discontinued treatment because of AEs or died before the first post‐baseline response assessment were included in the response analysis and counted as non‐responders to prevent overestimation. AEs that emerged within 28 days after the last treatment dose and before subsequent therapy were identified and graded according to the National Cancer Institute Common Terminology Criteria for Adverse Events (version 4.03). The AE categories included in this study have identical grade definitions in version 5.0; therefore, applying version 5.0 criteria does not alter grade assignments or safety comparisons.

### 2.5 Propensity‐Score Matching

To account for baseline differences between patients treated with PV‐R‐CHP and R‐CHOP, propensity‐score matching (PSM) [25] was performed. The variables were matched for age, sex, Ann Arbor Stage, Eastern Cooperative Oncology Group performance status (ECOG PS) ≥ 2, elevated serum lactate dehydrogenase (LDH), and presence of extra‐nodal disease ≥ 2. To adjust for potential confounders, we performed 1:1 propensity score matching using the nearest‐neighbor algorithm. A caliper width of 0.2 standard deviations of the logit‐transformed score was applied; unmatched patients were excluded if no acceptable match could be identified.

### 2.6 Statistical Analyses

Pearson's chi‐square test or Fisher's exact test was conducted to compare categorical covariables between the R‐CHOP and PV‐R‐CHP groups. The Mann–Whitney *U*‐test was used to determine significant differences in continuous baseline characteristics between the two groups. The probabilities of OS and PFS were estimated using the Kaplan–Meier method, with the log‐rank test performed to compare survival curves. The event rates at specific time points (e.g., 12‐month PFS/OS) were calculated along with 95% CIs and are reported as descriptive early estimates given the relatively short follow‐up; they were not intended to substitute the time‐to‐event primary endpoint. The Cox proportional hazards model was used to estimate the HRs and associated 95% CIs, as well as analyze the factors correlated with survival. The overall response rate (ORR) was calculated as the percentage of patients with complete or partial response. Differences with *p* < 0.05 were considered statistically significant. For dose‐intensity analyses, the propensity score‐matched cohort was designated as the analysis set to minimize confounding arising from baseline imbalances. This approach was adopted owing to the sensitivity of tARDI to patient mix. All statistical analyses were performed with EZR 1.60 (Saitama Medical Center, Jichi Medical University, Saitama, Japan) on R version 4.2.1 (R Foundation for Statistical Computing) [26].

## 3 Results

### 3.1 Patient Population

In total, 156 patients with DLBCL who received initial therapy across 3 years and 8 months were identified at our institution. Fifty‐six patients were treated with PV‐R‐CHP from September 1, 2022 to August 31, 2023. One hundred patients were treated with R‐CHOP or THP‐COP from January 1, 2020 to August 31, 2022 (Figures S1, S2). Three patients in the PV‐R‐CHP group were excluded as one patient discontinued treatment for personal reasons, one transferred, and one switched treatment after a revised diagnosis of high‐grade lymphoma, leaving 153 patients for the overall analysis. PSM was subsequently performed, yielding 50 patients per group in the matched cohort. Baseline characteristics are detailed in Table 1. The median (range) ages of patients treated with PV‐R‐CHP and R‐CHOP were similar at 72 (36–88) years and 75 (39–88) years, respectively. The proportion of patients aged > 80 years was 24.5% (13/53) and 32.0% (32/100) in the PV‐R‐CHP and R‐CHOP groups, respectively. Patients with IPI score ≥ 4–5 comprised 28.3% (15/53) of the PV‐R‐CHP group and 33.0% (33/100) of the R‐CHOP group. Patients with advanced Ann Arbor stage III‐IV comprised 52.8% and 54.0% of the PV‐R‐CHP and R‐CHOP groups, respectively. No significant differences were noted in the distribution of DLBCL COO (*p* = 0.907): germinal center B‐cell‐like (GCB), 49.1% and 45.0%; non‐GCB, 41.5% and 46.0%; unknown, 9.4% and 9.0% (PV‐R‐CHP and R‐CHOP, respectively). Cases transformed from follicular lymphoma were rare and similarly represented between groups (PV‐R‐CHP, *n* = 5; R‐CHOP, *n* = 7); one additional case transformed from another indolent lymphoma was included in the PV‐R‐CHP group. Although 27 patients (8 from the PV‐R‐CHP group and 19 from the R‐CHOP group) were excluded from the TMTV analysis due to the absence of PET‐CT‐evaluable lesions prior to treatment initiation, the median TMTV in the PV‐R‐CHP group was higher than that in the R‐CHOP group (*p* = 0.012). In the R‐CHOP group, 35 of 100 patients received R‐THP‐COP, and most of these individuals (25 of 35 [71.4%]) switched from DXR to pirarubicin primarily owing to older age (≥ 80 years) (Table 1). The remaining cases were treated with R‐THP‐COP owing to poor PS. No unfit patients received R‐THP‐COP after PV‐R‐CHP treatment approval.

**TABLE 1:** Baseline characteristics of patients treated with PV‐R‐CHP or R‐CHOP‐based regimens.

Characteristic	PV‐R‐CHP (*n* = 53)	R‐CHOP (*n* = 100)	*p* value	R‐CHOP only (*n* = 65)	*p* value versus PV‐R‐CHP
Age (years), median (range)	72.0 (36–88)	75.0 (39–88)	0.193	70.0 (39–87)	0.133
Age > 80 years, *n* (%)	13 (24.5)	32 (32.0)	0.358	9 (13.8)	0.159
Male, *n* (%)	31 (58.5)	55 (55.0)	0.734	35 (53.8)	0.710
IPI, *n* (%)			0.251		0.106
0	0 (0.0)	8 (8.0)		8 (12.3)	
1	15 (28.3)	18 (18.0)		11 (16.9)	
2	10 (18.9)	20 (20.0)		14 (21.5)	
3	13 (24.5)	21 (21.0)		15 (23.0)	
4	8 (15.1)	18 (18.0)		9 (13.8)	
5	7 (13.2)	15 (15.0)		8 (12.3)	
Ann Arbor stage, *n* (%)			0.893		0.762
I	8 (15.1)	18 (18.0)		14 (21.5)	
II	17 (32.1)	28 (28.0)		19 (29.2)	
III	8 (15.1)	13 (13.0)		7 (10.8)	
IV	20 (37.7)	41 (41.0)		25 (38.5)	
LDH (IU/L), median (range)	247.0 (149–2007)	248.0 (126–6719)	0.250	247 (126–6719)	0.271
≥ ULN, *n* (%)	29 (54.7)	59 (59.0)	0.612	28 (43.1)	0.207
≥ 2 × ULN, *n* (%)	10 (18.9)	21 (21.0)	0.835	10 (15.4)	0.631
ECOG PS ≥ 2, *n* (%)	22 (41.5)	48 (48.0)	0.575	27 (41.5)	1
Extra‐nodal lesion ≥ 2, *n* (%)	17 (31.1)	27 (27.0)	0.497	14 (21.5)	0.214
sIL‐2R (U/mL), median (range)	1206.0 (226–24,698)	1155.5 (49–37,358)	0.801	1077 (49–27,673)	0.498
Bulky lesion, *n* (%)	25 (47.2)	48 (48.0)	1.000	33 (50.8)	0.715
Cell of origin, *n* (%)			0.907		1
GCB	26 (49.1)	45 (45.0)		32 (49.2)	
Non‐GCB	22 (41.5)	46 (46.0)		26 (40.0)	
Unclassified	5 (9.4)	9 (9.0)		7 (10.8)	
Transformed from indolent lymphomas	6 (11.3)	7 (7.0)		5 (7.7)	0.539
Intrathecal prophylaxis	5 (9.4)	7 (7)	0.753	6 (9.2)	1
Total metabolic tumor volume			0.012		0.34
Median [Q1–Q3] (cm^3^)	94.7 [33.0–191.5] (*n* = 45)	31.6 [19.6–101.4] (*n* = 81)		78.9 [22.1**–**196.4] (*n* = 53)	

*Note:* The R‐CHOP group includes patients treated with either R‐CHOP or R‐THP‐COP. The R‐CHOP only group consists of patients treated with R‐CHOP but not R‐THP‐COP.

Abbreviations: ECOG PS, Eastern Cooperative Oncology Group performance status; GCB, germinal center B‐cell‐like; IPI, International Prognostic Index; LDH, lactate dehydrogenase; Q, quartile; sIL‐2R, soluble interleukin‐2 receptor; ULN, upper limit of normal.

### 3.2 Response to Treatment and Outcome

**FIGURE 1:**
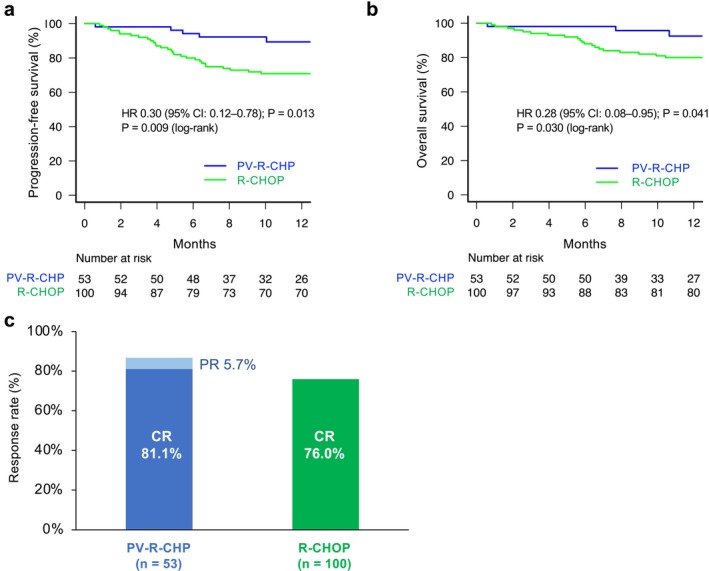
Efficacy outcomes for patients treated with PV‐R‐CHP or R‐CHOP‐based regimens in the overall, unmatched cohort. The median follow‐up time was 12.4 and 29.3 months in the PV‐R‐CHP and R‐CHOP groups, respectively. Progression‐free survival (a), overall survival (b), and response at the end of treatment (c).

The median follow‐up time was 373 (18–540) days and 878 (25–1505) days in the PV‐R‐CHP and R‐CHOP groups, respectively. The PFS of the PV‐R‐CHP group was significantly longer than that of the R‐CHOP group (HR 0.30 [95% CI: 0.12–0.78]; *p* = 0.013). The OS was also improved in the PV‐R‐CHP group (HR 0.28 [95% CI 0.08–0.95]; *p* = 0.041); however, this should be interpreted with caution given the limited follow‐up. At 1 year, the estimated PFS and OS were 89.3% versus 70.9% and 92.5% versus 80.0% (PV‐R‐CHP vs. R‐CHOP), respectively (Figure 1, Table S1). These 1‐year values are presented as early descriptive estimates, given the limited follow‐up. In sensitivity analyses, PV‐R‐CHP maintained significantly superior PFS and OS when follow‐up was truncated at 365 days (*p* = 0.016 and *p* = 0.048, respectively), demonstrating that the early survival advantage was not attributable to follow‐up imbalance. In a 12‐month landmark analysis among patients alive at 12 months after treatment initiation, no significant difference in landmark OS was observed (*p* = 1.00). PFS in the PV‐R‐CHP group was significantly higher than in the R‐CHOP group, even after excluding patients treated with THP‐COP (*p* = 0.026). Similarly, after excluding patients treated with R‐THP‐COP, the 1‐year OS was better in the PV‐R‐CHP group than in the R‐CHOP group, although not significantly (*p* = 0.137). Although patients with a very good R‐IPI (IPI score 0) were absent in the PV‐R‐CHP group, the R‐IPI did not distinguish good (IPI score 1, 2) and poor prognostic (IPI score 3–5) PV‐R‐CHP groups in terms of PFS and OS. Conversely, the R‐IPI distinguished three prognostic R‐CHOP groups based on PFS and OS (Figure S3). Among patients aged > 80 years, PFS and OS in the PV‐R‐CHP group exceeded those in the R‐CHOP group, albeit not significantly (data not shown). The ORR in PV‐R‐CHP‐treated patients was comparable with that of patients treated with R‐CHOP (86.8% vs. 76.0%, *p* = 0.141). The percentage of patients achieving complete response (CR) was similar between both groups (81.1% vs. 76.0%, *p* = 0.543) (Figure 1c). CNS relapse was observed in 1 (1.9%) and 6 (6.0%) patients in the PV‐R‐CHP and R‐CHOP groups, respectively. CNS relapse was observed only in patients with intermediate‐ or high‐risk CNS‐IPI. Among intermediate‐risk patients, relapse occurred in 0 of 23 (0%) in the PV‐R‐CHP group and 3 of 41 (7.3%) in the R‐CHOP group. Among high‐risk patients, relapse occurred in 1 of 15 (6.7%) and 3 of 33 (9.1%), respectively. Prophylactic intrathecal therapy was used infrequently in both groups across CNS‐IPI categories.

According to the analysis by COO category, no significant difference in PFS was observed between patients with GCB and non‐GCB, regardless of treatment regimen (PV‐R‐CHP, HR 1.23 [95% CI: 0.17–8.77]; *p* = 0.834, 1‐year PFS: 90.4% and 90.5%; R‐CHOP, HR 1.57 [95% CI: 0.76–3.26]; *p* = 0.225, 1‐year PFS: 75.6% and 65.2%) (Figure S4a). Overall survival for patients with GCB and non‐GCB DLBCL was comparable in both the PV‐R‐CHP and R‐CHOP groups (PV‐R‐CHP, HR 1.30 [95% CI: 0.080–21.36]; *p* = 0.853, 1‐year OS: 94.7% and 90.0%; R‐CHOP, HR 2.00 [95% CI: 0.89–4.49]; *p* = 0.093, 1‐year OS: 82.2% and 76.1%) (Figure S4b). Similarly, in the PV‐R‐CHP group, PFS was comparable between patients with IPI scores 0–1 and 2–5 (Figure S5a). Overall survival in the PV‐R‐CHP group was not comparable between patients with IPI scores 0–1 and 2–5 because all patients with IPI score 0–1 were alive (Figure S5b). Although a statistical comparison of the HR in the R‐CHOP group was not feasible since all patients with IPI score 0–1 were alive at analysis, patients with IPI scores 2–5 had inferior PFS and OS compared to those with scores 0–1 (Figure S5). Elevated LDH and bulky lesions were associated with worse outcomes, particularly in the R‐CHOP group, where significant differences were observed in PFS and OS (Figures S6 and S7). CR rates did not differ significantly across subgroups (data not shown).

The univariate analysis showed that PV‐R‐CHP treatment was associated with improved PFS, while worse PFS was significantly linked to Ann Arbor stage ≥ III, elevated LDH, bulky lesions, and elevated soluble interleukin‐2 receptor (sIL‐2R). In addition to these risk factors, age > 80 years was pinpointed as a risk factor for worse OS. In the multivariate analysis, PV‐R‐CHP treatment emerged as a favorable factor for PFS, whereas worse OS was significantly associated with age > 80 years, Ann Arbor stage ≥ III, and bulky lesions (Table 2).

**TABLE 2:** Univariate and multivariate analyses for progression‐free and overall survival.

	Progression‐free survival				Overall survival			
	Univariate analysis		Multivariate analysis		Univariate analysis		Multivariate analysis	
	HR (95% CI)	*p* value	HR (95% CI)	*p* value	HR (95% CI)	*p* value	HR (95% CI)	*p* value
PV‐R‐CHP	0.31 (0.12–0.78)	0.013	0.36 (0.14–0.94)	0.040	0.28 (0.08–0.95)	0.041	0.39 (0.11–1.33)	0.131
Age > 80 years	1.79 (0.93–3.43)	0.079	1.64 (0.84–3.21)	0.147	2.38 (1.17–4.82)	0.017	2.65 (1.27–5.53)	0.010
Male	1.29 (0.67–2.46)	0.450	—	—	1.52 (0.73–3.17)	0.267	—	—
Ann Arbor Stage III–IV	1.84 (1.31–2.58)	< 0.001	1.47 (1.00–2.16)	0.050	2.01 (1.36–2.98)	< 0.001	1.72 (1.09–2.71)	0.019
LDH ≥ ULN	6.23 (2.43–15.97)	< 0.001	2.40 (0.77–7.49)	0.131	6.17 (2.16–17.65)	< 0.001	2.13 (0.61–7.33)	0.238
Bulky lesion (+)	3.19 (1.58–6.44)	0.012	1.83 (0.84–3.99)	0.126	3.76 (1.68–8.41)	0.001	2.53 (1.05–6.05)	0.038
Non‐GCB	1.35 (0.84–2.18)	0.211	—	—	1.44 (0.86–2.41)	0.170	—	—
DEL	1.45 (0.944–2.22)	0.090	—	—	1.54 (0.96–2.48)	0.073	—	—
sIL‐2R > 1170 U/mL (median)	3.51 (1.70–7.22)	< 0.001	1.40 (0.61–3.17)	0.427	2.90 (1.33–6.30)	0.007	1.01 (0.42–2.40)	0.984

Abbreviations: CI, confidence interval; HR, hazard ratio; ULN, upper limit of normal.

### 3.3 PSM Analysis

One hundred patients treated with PV‐R‐CHP or R‐CHOP (50 patients in each group) were included after PSM (Table S2). In the R‐CHOP group, 15 patients treated with R‐THP‐COP were included. The following analyses were conducted in the propensity‐matched cohorts.

The median number of received cycles was six (range, 1–6) for patients in each group (Table S3). The completion rates of the 6 cycles were 86.0% (43/50) and 66.0% (33/50) for the PV‐R‐CHP and R‐CHOP groups, respectively. Seven and 17 patients discontinued the treatment prior to completing 6 cycles in the PV‐R‐CHP and R‐CHOP groups, respectively. The reasons for treatment discontinuation are tabulated in Table S3. The other reasons for treatment discontinuation in the R‐CHOP group included death of unknown cause in two patients, complication of lung cancer in one patient, and the patient's request in one patient. The median (range) tARDI was 78.0% (24.0%–100%) in PV‐R‐CHP‐treated patients and 64.0% (3.0%–100%) in R‐CHOP‐treated patients (*p* = 0.008). The median (range) RDI for PV was 88.0% (33.0%–100.0%), with only one case of dose reduction. The median (range) RDI for VCR was 50.0% (0.0%–100.0%), and VCR was either not administered or discontinued/reduced in 82.0% (41/50) of patients. The median tARDI among patients aged > 80 years was 60% in the PV‐R‐CHP group (*n* = 13) versus 48% in the R‐CHOP group (*n* = 15; *p* = 0.076) (data not shown).

**FIGURE 2:**
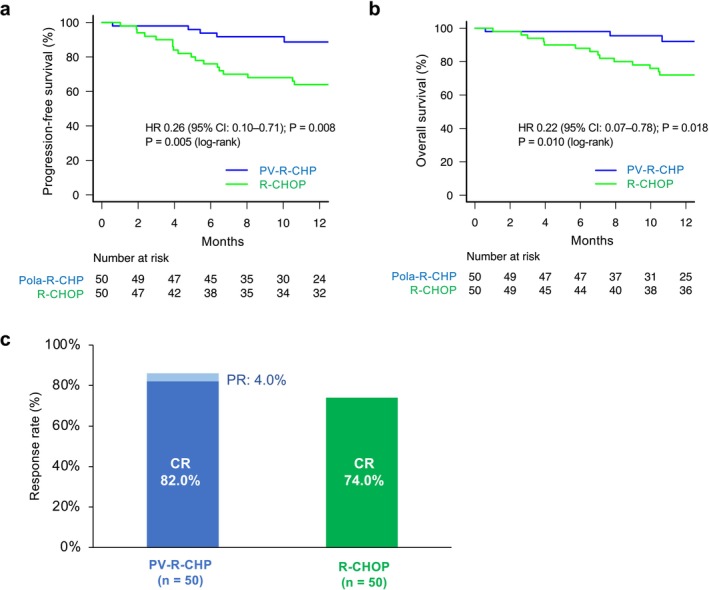
Efficacy outcomes for patients treated with PV‐R‐CHP or R‐CHOP‐based regimens after propensity score matching. The median follow‐up time was 12.3 and 28.1 months in the PV‐R‐CHP and R‐CHOP groups, respectively. Progression‐free survival (a), overall survival (b), and response at the end of treatment (c). CR, complete response; PR, partial response.

The PFS of the PV‐R‐CHP group was significantly longer than that of the R‐CHOP group (HR 0.26 [95% CI: 0.10–0.71]; *p* = 0.008). Likewise, the PV‐R‐CHP group exhibited favorable OS (HR 0.22 [95% CI: 0.06–0.78]; *p* = 0.018). At 1 year, the estimated PFS was 88.7% versus 64.0% and the estimated OS was 92.1% versus 72.0% (PV‐R‐CHP vs. R‐CHOP; 1‐year values were interpreted as descriptive, as noted earlier) (Table S1, Figure 2). The most frequent cause of mortality in the treatment groups was disease progression (PV‐R‐CHP: 2, R‐CHOP: 12). Infectious causes of death were reported in one and two cases in the PV‐R‐CHP and R‐CHOP groups, respectively. One patient died of lung cancer in the R‐CHOP group. Two patients whose deaths were attributed to unknown causes were from the R‐CHOP group. The ORR at the end of treatment was comparable between PV‐R‐CHP‐treated patients and R‐CHOP‐treated patients (86.0% vs. 74.0%, *p* = 0.211). The percentage of CR was similar between the PV‐R‐CHP and R‐CHOP (82.0% vs. 74.0%, *p* = 0.470) groups.

**FIGURE 3:**
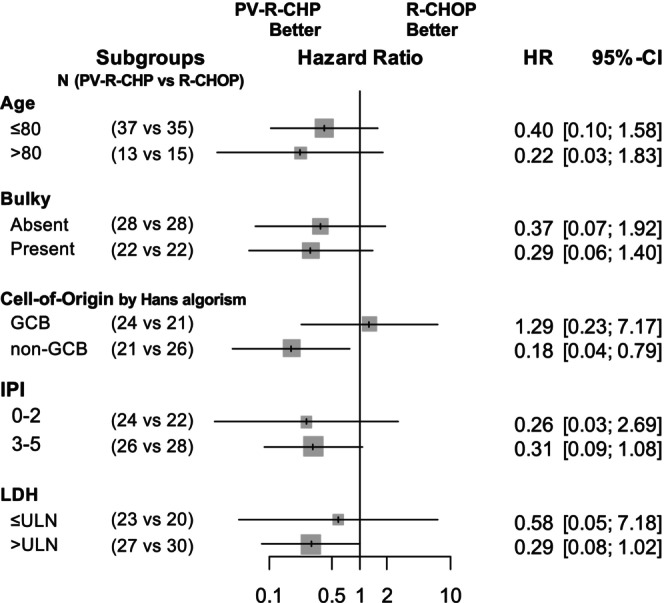
Forest plot for progression‐free survival based on clinical and biological characteristics in the propensity score‐matched cohort. DEL, double‐expressor lymphoma; GCB, germinal center B‐cell‐like; IHC, immunohistochemistry; IPI, International Prognostic Index; LDH, lactate dehydrogenase; NA, not available; ULN, upper limit of normal.

The results of the subgroup analysis of PFS are shown in Figure 3. PV‐R‐CHP demonstrated a significant survival benefit in patients with non‐GCB DLBCL. Although PV‐R‐CHP showed improved outcomes in patients with IPI score 3–5 and LDH elevation, these differences did not reach statistical significance. No other subgroups indicated the superiority of PV‐R‐CHP over R‐CHOP, whereas no subgroups demonstrated a clear benefit with R‐CHOP.

### 3.4 Safety

The safety profiles of patients treated with PV‐R‐CHP and R‐CHOP were largely comparable, with similar AE types, frequencies, and grades observed in both groups (Table 3 and Figure S8). The most prevalent grade 3 or 4 AE was lymphopenia, occurring in 48.0% of PV‐R‐CHP‐treated patients and 52.0% of R‐CHOP‐treated patients. Grade 3 or 4 neutropenia was observed in 38.0% of PV‐R‐CHP‐treated patients and in 52.0% of R‐CHOP‐treated patients. Grade 3 or 4 neutropenia occurred in 38.0% of PV‐R‐CHP‐treated patients and 52.0% of R‐CHOP‐treated patients. Pegfilgrastim was administered prophylactically in all PV‐R‐CHP‐treated patients, whereas six of 50 R‐CHOP‐treated patients did not receive pegfilgrastim. Patients with Grade 3 or 4 infections comprised 28% and 36% of the PV‐R‐CHP and R‐CHOP groups, respectively. The most common infectious events of any grade, except for coronavirus disease 2019 (COVID‐19), were colitis (Grade 2: 6%) in the PV‐R‐CHP group and febrile neutropenia (Grade 3: 18%) in the R‐CHOP group. The incidence rate of febrile neutropenia in the PV‐R‐CHP group was 4% (Grade 3). The incidence of COVID‐19 infection was 24% and 2% in the PV‐R‐CHP and R‐CHOP groups, respectively. Eight of the 12 patients (66.7%) with COVID‐19 in the PV‐R‐CHP group required hospitalization owing to Grade 3–4 severity, with no associated deaths. Discrepancies were observed in the prevalence of COVID‐19 between PV‐R‐CHP and R‐CHOP groups (24% vs. 2%). The higher incidence in the PV‐R‐CHP group is likely attributable to the COVID‐19 outbreak periods in Japan. Grade 5 AEs leading to death were observed in one patient and two patients from the PV‐R‐CHP and R‐CHOP groups, respectively. These events were primarily related to infections, including sepsis in one PV‐R‐CHP‐treated patient and pneumonia in two R‐CHOP‐treated patients.

**TABLE 3:** Adverse events in propensity score‐matched populations.

Adverse event	PV‐R‐CHP		R‐CHOP		*p* value (any grade)	*p* value (grade 3–4)
	Any grade, *n* (%)	Grade 3–4, *n* (%)	Any grade, *n* (%)	Grade 3–4, *n* (%)		
Neutropenia	25 (50)	19 (38)	31 (62)	26 (52)	0.31	0.23
Lymphopenia	40 (80)	24 (48)	40 (80)	26 (52)	1	0.84
Anemia	37 (74)	6 (12)	40 (80)	4 (8)	0.64	0.74
Thrombocytopenia	25 (50)	8 (16)	30 (60)	6 (12)	0.42	0.77
Liver/Hepatic disorder	13 (26)	0 (0)	18 (36)	1 (2)	0.39	—
Peripheral neuropathy	13 (26)	1 (2)	21 (42)	3 (6)	0.14	0.62
Constipation	32 (64)	2 (4)	36 (72)	0 (0)	0.52	0.50
Diarrhea	10 (20)	0 (0)	1 (2)	0 (0)	< 0.01	—
Fatigue	8 (16)	3 (6)	4 (8)	2 (4)	—	—
Infusion reaction	4 (8)	0 (0)	5 (10)	0 (0)	—	—
Infection	11 (22)	4 (8)	10 (20)	8 (16)	0.84	0.36
Febrile neutropenia	—	2 (4)	—	9 (18)	—	0.05
COVID‐19	12 (24)	8 (16)	1 (2)	1 (2)	< 0.01	0.03
Other	5 (10)	3 (6)	7 (14)	6 (12)	—	—

*Note:* The R‐CHOP group includes patients treated with either R‐CHOP or R‐THP‐COP.

Peripheral neuropathy (PN) was observed in 26% (2% at grade 3–4) and 42% (6% at grade 3–4) of patients who received PV‐R‐CHP and R‐CHOP, respectively. Constipation incidence at any grade was marginally lower in PV‐R‐CHP‐treated patients (64%) than in R‐CHOP‐treated patients (72%). A transient incidence of mild diarrhea (Grade 1–2) was observed in 20% of PV‐R‐CHP‐treated patients; however, it was rare in R‐CHOP‐treated patients.

## 4 Discussion

In this retrospective study, we evaluated the efficacy and safety of PV‐R‐CHP compared with those of R‐CHOP in real‐world settings, which has remained unknown thus far. The results demonstrated a significant survival benefit of PV‐R‐CHP compared with R‐CHOP for previously untreated DLBCL. The safety profiles of PV‐R‐CHP and R‐CHOP were similar.

In the POLARIX trial, the 2‐year PFS in patients treated with PV‐R‐CHP was significantly higher than in those treated with R‐CHOP, with a minimal difference (6.5%) that could be meaningful for long‐term prognosis in patients with DLBCL.

Twenty‐four months after receiving the initial rituximab‐containing anthracycline‐based immunochemotherapy, patients without progression may display survival outcomes similar to those of the general population [27, 28]. In this study, patients treated with PV‐R‐CHP have better OS and PFS compared to those treated with R‐CHOP, despite the short follow‐up period. We believe the early (1‐year) treatment results will guide clinical practice because novel treatments, such as chimeric antigen receptor T‐cell and bispecific antibody, are recently available as a standard of care in Japan. PV‐R‐CHP treatment was significantly associated with favorable PFS, but not with OS, in the multivariate analysis, thus indicating the relatively limited impact of first‐line treatment on OS in short‐term follow‐up because some patients who relapse or are refractory to first‐line treatment respond to salvage therapies. Considering the short follow‐up in the PV‐R‐CHP group, late relapses and toxicities may have been overlooked, and this limitation particularly affects the interpretation of OS. The absence of a significant effect on OS in the multivariate analysis should therefore be carefully interpreted until longer follow‐up is available. Sensitivity analyses yielded similar conclusions, indicating that the observed early advantage of PV‐R‐CHP was not solely due to differences in follow‐up. We reported results for the entire cohort to provide a comprehensive view of the study population. Because this study reflects a real‐world setting, presenting data that reflect the broader clinical context—including patients with varying baseline characteristics and risk factors—offers important insights. Although the PSM analysis minimizes baseline imbalances and provides a more controlled comparison, presenting the entire cohort results allows readers to understand outcomes as they occur in routine clinical practice. We believe this dual approach enhances the generalizability and applicability of our findings.

Approximately 50% of patients had non‐GCB type, with 31% having activated B‐cell‐like (ABC) type in the POLARIX study. Although a higher percentage of ABC DLBCL was observed in Asian countries, a disparity between the non‐GCB type in this study and ABC type in the POLARIX study may exist, possibly owing to the Hans algorithm used for COO classification. In the overall cohort, no significant differences in PFS and OS were observed between GCB and non‐GCB subgroups in both treatment groups. According to the PSM analysis, the PFS was longer in the non‐GCB subgroup of PV‐R‐CHP compared with that in the R‐CHOP. However, given the limited sample size and the exploratory nature of subgroup analyses, this finding should be considered hypothesis‐generating rather than conclusive. Although formal comparative analyses were not conducted within GCB and non‐GCB subgroups, both groups exhibited a higher probability of improving PFS with PV‐R‐CHP than with R‐CHOP irrespective of the COO in the overall cohort (Figure S4). Although previous studies have demonstrated the efficacy of PV in ABC DLBCL [15, 16], our findings suggest that the therapeutic benefit of PV‐R‐CHP may not be restricted to ABC DLBCL. Additionally, a post hoc analysis following the POLARIX study revealed that PV‐R‐CHP may improve 2‐year PFS compared with R‐CHOP in patients with GCB‐type DLBCL who exhibited a dark zone gene expression signature [29].

PV‐R‐CHP may be beneficial to patients with IPI scores 3–5. In this study, the R‐IPI score probably does not apply to patients treated with PV‐R‐CHP; however, it is applicable to others treated with R‐CHOP. Developing a new prognostic score may be necessary for patients treated with PV‐R‐CHP. In Japan, unlike in many other countries, PV‐R‐CHP treatment is also available to patients with IPI scores of 0 or 1 who were not eligible for the POLARIX study and is covered by public health insurance. In patients with IPI scores 0 or 1, the PFS of those treated with PV‐R‐CHP and R‐CHOP was markedly high, and no notable benefit of PV‐R‐CHP was observed. However, a longer observation period may be necessary to determine PV‐R‐CHP efficacy in these cases as it demonstrates a longer response duration. Notably, the POLARIX study reported superior disease‐free survival and duration of response by PV‐R‐CHP, whereas ORR was comparable between PV‐R‐CHP and R‐CHOP [16].

The median age was 73 years, approximately 8 years more than that of the POLARIX trial patient population, and included patients > 80 years old who are ineligible for POLARIX. For most patients ≥ 80 years old, an attenuated chemoimmunotherapy, such as R‐miniCHOP, is generally used to avoid toxicity [30], despite its inferior PFS and OS compared with those of R‐CHOP [31]. Although the clinical efficacy and safety of PV‐R‐miniCHP for patients > 80 years old remain unknown, preliminary evidence suggests that this regimen is equally tolerable as R‐miniCHP [32]. In this study, the median RDI for PV was higher than that of other agents except for rituximab, indicating higher tolerability of PV. Monomethyl auristatin E, a cytotoxic agent used in PV, has high cytotoxic potential against lymphoma cells [33].

PV may enhance antitumor activity in combination with rituximab by elevating CD20 expression of lymphoma cells [34]. Therefore, utilizing PV‐R‐miniCHP, a treatment involving PV at standard dose, instead of reduced VCR, may be more effective and equally tolerable compared with R‐miniCHP for patients > 80 years old.

PV‐R‐CHP was generally well tolerated in this study, with manageable toxicities. The incidence of neutropenia, anemia, and thrombocytopenia was similar between treatment regimens. Anemia was common (~80% for any grade) in both groups, potentially due to the higher proportion of older patients. Consistent with the POLARIX study findings, neutropenia incidence was higher in PV‐R‐CHP‐treated patients than in R‐CHOP‐treated patients. The lower incidence of infection observed in the PV‐R‐CHP group may partly reflect the widespread use of prophylactic pegfilgrastim in our institution. However, pegfilgrastim coverage was similarly high in the PSM cohorts (100% vs. 88%); therefore, this difference is unlikely to fully explain the finding, and causality cannot be inferred. Although prophylactic pegfilgrastim use was nearly universal in both regimens, infection‐related outcomes may still reflect era‐specific factors, including higher COVID‐19 incidence during the period when PV‐R‐CHP was exclusively used. These factors are inherently linked to the treatment era and should be interpreted as potential residual confounders. PV‐R‐CHP‐treated patients had a lower incidence of any grade PN (26%) in this study than in the POLARIX study (53%). Furthermore, PN incidence in patients treated with PV‐R‐CHP was lower than that in patients treated with R‐CHOP. Similarly, the Asian subpopulation analysis of the POLARIX study showed a lower PN incidence (44%) in patients treated with PV‐R‐CHP than it did in the global POLARIX population [35]. These results may suggest that Asian patients are less susceptible to PV‐induced PN.

Regarding VCR, the dose reduction rate was high in this study despite the relatively lower PN incidence. We usually reduce the dose of CHOP or use miniCHOP from the first cycle for older and frail patients. Furthermore, we excluded VCR administration for patients with extensive mesenteric disease or prior abdominal surgery to prevent severe constipation or ileus. The R‐CHOP group included 11 patients with mesenteric disease after matching the cohort. This strategy may contribute to the high rate of VCR dose reduction in this study despite the relatively lower PN incidence. In addition, the higher apparent rate of VCR dose reduction in the R‐CHOP group may be partially explained by the inclusion of R‐THP‐COP, which uses a standard VCR dose of 1.0 mg/m^2^ rather than the higher standard dose of 1.4 mg/m^2^ used in R‐CHOP. Moreover, the better tolerability of PV, especially with respect to PN, may have necessitated fewer dose modifications. The inferior outcomes observed in the R‐CHOP group may, in part, be attributable to the dose reduction of VCR.

Several real‐world studies evaluating frontline PV‐R‐CHP have been reported recently. For example, Zhao et al. conducted a retrospective study in China and found no significant difference in PFS between patients treated with PV‐R‐CHP and those treated with R‐CHOP [36]. Notably, survival in their R‐CHOP cohort appeared more favorable than typically reported in pivotal trials, which may have attenuated between‐group differences. In contrast, in our Japanese cohort, which was older on average and reflected local clinical practices (e.g., inclusion of R‐THP‐COP in the comparator arm, prophylactic pegfilgrastim, and no post‐cycle rituximab), PV‐R‐CHP was associated with improved early PFS and OS compared to R‐CHOP‐based regimens. We also performed PSM and detailed dose intensity and dose modifications (including VCR and PV adjustments). The differences in baseline characteristics and practice patterns may have contributed to the divergent findings. Nonetheless, our single‐center design and relatively short follow‐up (12 months), which is comparable to other real‐world PV‐R‐CHP reports, are important limitations; longer observation is needed to confirm durability and benchmark against 24‐month endpoints reported in pivotal trials.

As a single‐center retrospective analysis, this study is subject to inherent limitations. First, the small number of patients, especially those treated with PV‐R‐CHP, may limit the statistical power to detect significant differences or draw definitive conclusions. Second, the follow‐up duration was shorter in the PV‐R‐CHP group than in the R‐CHOP group, reflecting their respective approval timelines. This relatively short observation period in the PV‐R‐CHP group, along with the difference in follow‐up between groups, may preclude the inclusion of late relapses and toxicities in the analysis and limit the ability to fully assess long‐term efficacy and safety. In particular, both PFS and OS outcomes should be interpreted with caution, as longer follow‐up is required to confirm the durability of the treatment benefit and identify late AEs.

Third, the known risk factors were included as variables in the propensity score analysis; however, some potentially unmeasured confounders could not be adjusted for. Additionally, some patients received R‐THP‐COP instead of R‐CHOP owing to advanced age, cardiac complications, or poor PS. Although this reflects real‐world practice, the inclusion of these patients may have introduced selection bias, potentially influencing the outcomes observed in the R‐CHOP group. At our institution, R‐THP‐COP was often preferred over reduced‐dose R‐CHOP for older or frail patients during the study period, which may explain the relatively high proportion of R‐THP‐COP cases in the control group. Furthermore, the subgroup analysis excluding patients treated with R‐THP‐COP was conducted to reduce confounding; however, the smaller sample size in this subgroup may limit the robustness of the findings. Finally, the impact of PV‐R‐CHP and R‐CHOP on quality of life was not assessed in this study. Despite these limitations, the study provided valuable real‐world data on PV‐R‐CHP. Finally, because our institution switched completely from R‐CHOP to PV‐R‐CHP in August 2022, treatment allocation was entirely determined by calendar time. Consequently, treatment effects cannot be fully separated from era‐related influences. Our findings should be interpreted as reflecting a combined effect of regimen and era, with possible residual confounding from temporal changes in diagnostics, supportive care, COVID‐19 impact, or availability of salvage therapies.

In conclusion, PV‐R‐CHP demonstrated PFS and OS benefits, with similar tolerability to R‐CHOP among untreated patients with DLBCL. Although further long‐term follow‐up and studies involving a larger population are required to confirm these results, the study findings will help to facilitate the clinical application of PV‐R‐CHP among patients with untreated DLBCL.

## Author Contributions

**Masaaki Hotta:** conceptualization, methodology, investigation, formal analysis, writing – original draft, data curation. **Atsushi Satake:** conceptualization, methodology, investigation, writing – original draft, review and editing, validation, project administration, supervision. **Ayako Iwama:** investigation. **Tokiko Hoshiyama:** investigation. **Yukie Tsubokura:** investigation. **Hideaki Yoshimura:** investigation. **Shinya Fujita:** investigation. **Yumiko Kono:** formal analysis. **Tomoki Ito:** investigation, supervision.

## Funding

This study received financial support from Kansai Medical University in Osaka, Japan.

## Ethics Statement

All research procedures conformed to the Declaration of Helsinki and were sanctioned by the institutional review board of the Faculty of Medicine, Kansai Medical University. Participants provided written informed consent through an opt‐out approach as detailed on the institution's website.

## Conflicts of Interest

Atsushi Satake received honoraria from Chugai. Tomoki Ito received honoraria from Bristol‐Myers Squibb, Novartis, Takeda, AbbVie, Chugai, Eisai, Sanofi, AstraZeneca, and Janssen. Tomoki Ito also received funds from Bristol‐Myers Squibb, Otsuka, Mochida, Asahi Kasei, Taisho, and Chugai. Other authors do not have conflicts of interest.

## Supporting information


**Table S1:** Summary of progression‐free and overall survival rates of patients treated with PV‐R‐CHP or R‐CHOP in the overall and propensity score‐matched cohorts.
**Table S2:** Baseline characteristics of propensity score‐matched populations.
**Table S3:** Treatment exposure in propensity‐score matched patients treated with PV‐R‐CHP or R‐CHOP‐based regimens.
**Figure S1:** Overview of patient inclusion and propensity‐score matching. The R‐CHOP group includes patients treated with either R‐CHOP or R‐THP‐COP.
**Figure S2:** Monthly distribution of PV‐R‐CHP and R‐CHOP‐based treatments.
**Figure S3:** Efficacy outcomes based on the revised International Prognostic Index for patients treated with PV‐R‐CHP or R‐CHOP‐based regimens in the overall, unmatched cohort. Progression‐free survival (a) and overall survival (b) in the PV‐R‐CHP group. Progression‐free survival (c) and overall survival (d) in the R‐CHOP group.
**Figure S4:** Survival outcomes for patients treated with PV‐R‐CHP or R‐CHOP‐based regimens based on the cell of origin in the overall, unmatched cohort. Progression‐free survival (a) and overall survival (b) in patients with germinal center B‐cell‐like (GCB) and non‐GCB diffuse large B‐cell lymphoma.
**Figure S5:** Survival outcomes for patients treated with PV‐R‐CHP or R‐CHOP‐based on the International Prognostic Index in the overall, unmatched cohort. Progression‐free survival (a) and overall survival (b).
**Figure S6:** Survival outcomes for patients treated with PV‐R‐CHP or R‐CHOP‐based on lactate dehydrogenase elevation in the overall, unmatched cohort. Progression‐free survival (a) and overall survival (b).
**Figure S7:** Survival outcomes of patients treated with PV‐R‐CHP or R‐CHOP‐based on the presence of a bulky mass lesion in the overall, unmatched cohort. Progression‐free survival (a) and overall survival (b).
**Figure S8:** Butterfly plot showing the proportion of adverse events in propensity score‐matched populations. The R‐CHOP group includes patients treated with either R‐CHOP or R‐THP‐COP.

## Data Availability

The data that support the findings of this study are available from the corresponding author upon reasonable request.
