# Supplementary material for: Real‐World Outcomes of Polatuzumab Vedotin Plus R‐CHP Versus R‐CHOP‐Based Regimens in Japanese Patients With Untreated Diffuse Large B‐Cell Lymphoma
Source: Cancer Med. 2026 Jan 12;15(1):e71531. doi: 10.1002/cam4.71531 (PMC12793779; doi:10.1002/cam4.71531)

**Table S1. Summary of progression-free and overall survival rates of patients treated with PV-R-CHP or R-CHOP-based regimens in overall and propensity score-matched cohorts**

|  | | **PV-R-CHP** | **R-CHOP** | **HR (95%CI)** | **P value** |
| --- | --- | --- | --- | --- | --- |
|  |  | **1 year (95% CI)** | |  |  |
| **PFS** | **Unmatched**  PV-R-CHP (n=53), R-CHOP (n=100) | 89.30%  (75.9–95.5) | 70.90%  (78.0–96.6) | 0.30  (0.12–0.78) | 0.013 |
|  | **PS matched**  PV-R-CHP (n=50), R-CHOP (n=50) | 88.70%  (74.5–95.2) | 64.00%  (49.1–75.6) | 0.26  (0.10–0.71) | 0.008 |
| **OS** | **Unmatched**  PV-R-CHP (n=53), R-CHOP (n=100) | 92.50%  (60.9–78.8) | 80.00%  (70.7–86.6) | 0.28  (0.09–0.95) | 0.041 |
|  | **PS matched**  PV-R-CHP (n=50), R-CHOP (n=50) | 92.10%  (76.8–97.5) | 72.00%  (57.4–82.4) | 0.22  (0.07–0.78) | 0.018 |

The R-CHOP group includes patients treated with either R-CHOP or R-THP-COP.

CI, confidence interval; PFS, progression-free survival; PS, propensity score

**Table S2. Baseline characteristics of propensity score-matched populations**

| **Characteristic** | **PV-R-CHP**  **(n=50)** | **R-CHOP/THP-COP**  **(n=35/15)** | **P value** |
| --- | --- | --- | --- |
| Age, median (range) | 73.0 (36–88) | 74.5 (39–88) | 0.341 |
| Age >80 years, n (%) | 13 (26) | 15 (30) | 0.824 |
| Male, n (%) | 28 (56) | 29 (58) | 1.000 |
| IPI, n (%) |  |  | 0.284 |
| 0 | 0 (0) | 3 (6) |  |
| 1 | 14 (28) | 8 (16) |  |
| 2 | 10 (20) | 11 (22) |  |
| 3 | 13 (26.0) | 11 (22) |  |
| 4 | 8 (16.0) | 7 (14) |  |
| 5 | 5 (10) | 10 (20) |  |
| Ann Arbor Stage, n (%) |  |  | 0.559 |
| 𝖨 | 8 (16) | 10 (20) |  |
| Ⅱ | 17 (34) | 11 (22) |  |
| Ⅲ | 6 (12) | 5 (10) |  |
| Ⅳ | 19 (38.0) | 24 (48) |  |
| LDH, median (range) | 238 (149–1,850) | 238 (126–1,753) | 0.945 |
| ≥ULN, n (%) | 27 (54) | 30 (60) |  |
| ≥2×ULN, n (%) | 9 (18) | 10 (20) |  |
| sIL-2R, median (range) | 1171 (226–24,698) | 1092 (230–27,673) | 0.609 |
| ECOG PS ≥2, n (%) | 19 (38) | 22 (44) | 0.685 |
| Extra-nodal lesion ≥2, n (%) | 14 (28.0) | 19 (38) | 0.395 |
| Bulky lesion, n (%) | 22 (44) | 22 (44) | 1.000 |
| Cell of origin, n (%) |  |  | 0.566 |
| GCB | 24 (48) | 21 (42) |  |
| Non-GCB | 21 (42) | 26 (52) |  |
| Unclassified | 5 (10) | 3 (6) |  |
| Transformed from indolent lymphomas, n(%) | 6 (12) | 5 (10) | 1 |
| DEL by IHC |  |  | 0.267 |
| DEL | 19 (38) | 26 (52) |  |
| Non-DEL | 20 (40) | 13 (26) |  |
| NA | 11 (22) | 11 (22) |  |
| Intrathecal prophylaxis | 5 (10) | 3 (6.0) | 0.715 |
| Total metabolic tumor volume |  |  | 0.682 |
| Median [Q1-Q3] (cm^3^) | **91.57** [35.84–191.78]  (n=42) | **28.10 [19.75–98.77]**  (n=40) | 0.018 |

IPI, International Prognostic Index; LDH, lactate dehydrogenase; ULN, upper limit of normal; sIL-2R, soluble interleukin-2 receptor; ECOG PS, Eastern Cooperative Oncology Group performance status; GCB, germinal center B-cell-like; DEL, double expressor lymphoma; IHC, immunohistochemistry; NA, not applicable.

**Table S3 Treatment exposure in propensity-score matched patients treated with PV-R-CHP or R-CHOP-based regimens**

| **Parameter** | **PV-R-CHP (n=50)** | **R-CHOP (n=50)** | **P value** |
| --- | --- | --- | --- |
| Median number of cycles completed (range) | 6 (1–6) | 6 (1–8) | 0.151 |
| Completed six cycles, n (%) | 43 (86.0) | 33 (66.0) | 0.034 |
| Reason for treatment discontinuation |  |  |  |
| Progressive disease, n (%) | 0 (0) | 4 (8.0) | 0.117 |
| Adverse events, n (%) | 4 (8.0) | 4 (8.0) | 1 |
| Limited stage, n (%) | 3 (6.0) | 5 (10.0) | 0.715 |
| Other, n (%) | 0 (0) | 4 (8.0) | 0.117 |
| Polatuzumab vedotin dose reduction, n (%) | 1 (2.0) | - | - |
| Vincristine dose reduction, n (%) | - | 41 (82.0) | - |
| Rituximab dose reduction, n (%) | 1 (2.0) | 0 | 1 |
| DXR/THP and CPA dose reduction, n (%) | 28 (56.0) | 32 (64.0) | 0.541 |
| Relative dose index (%), median (range) |  |  |  |
| Polatuzumab vedotin | 88.0 (33.0–100.0) | - | - |
| Vincristine | - | 50.0 (0.0–100.0) | - |
| Rituximab | 88.0 (33.0–100.0) | 91.0 (51.0–120.0) | 0.504 |
| Cyclophosphamide | 72.0 (8.0–100.0) | 65.0 (30.0–100.0) | 0.588 |
| DXR/THP | 72.0 (8.0–100.0) | 63.0 (0.0–100.0) | 0.488 |
| tARDI (%), Median (range) | 78.0 (24.0–100.0) | 64.0 (3.0–100.0) | 0.008 |

The R-CHOP group includes patients treated with either R-CHOP or R-THP-COP.

CPA, cyclophosphamide; DXR, doxorubicin; tARDI, total average relative dose intensity; THP, pirarubicin.

**Figure S1 Overview of patient inclusion and propensity-score matching.** The R-CHOP group includes patients treated with either R-CHOP or R-THP-COP.

**
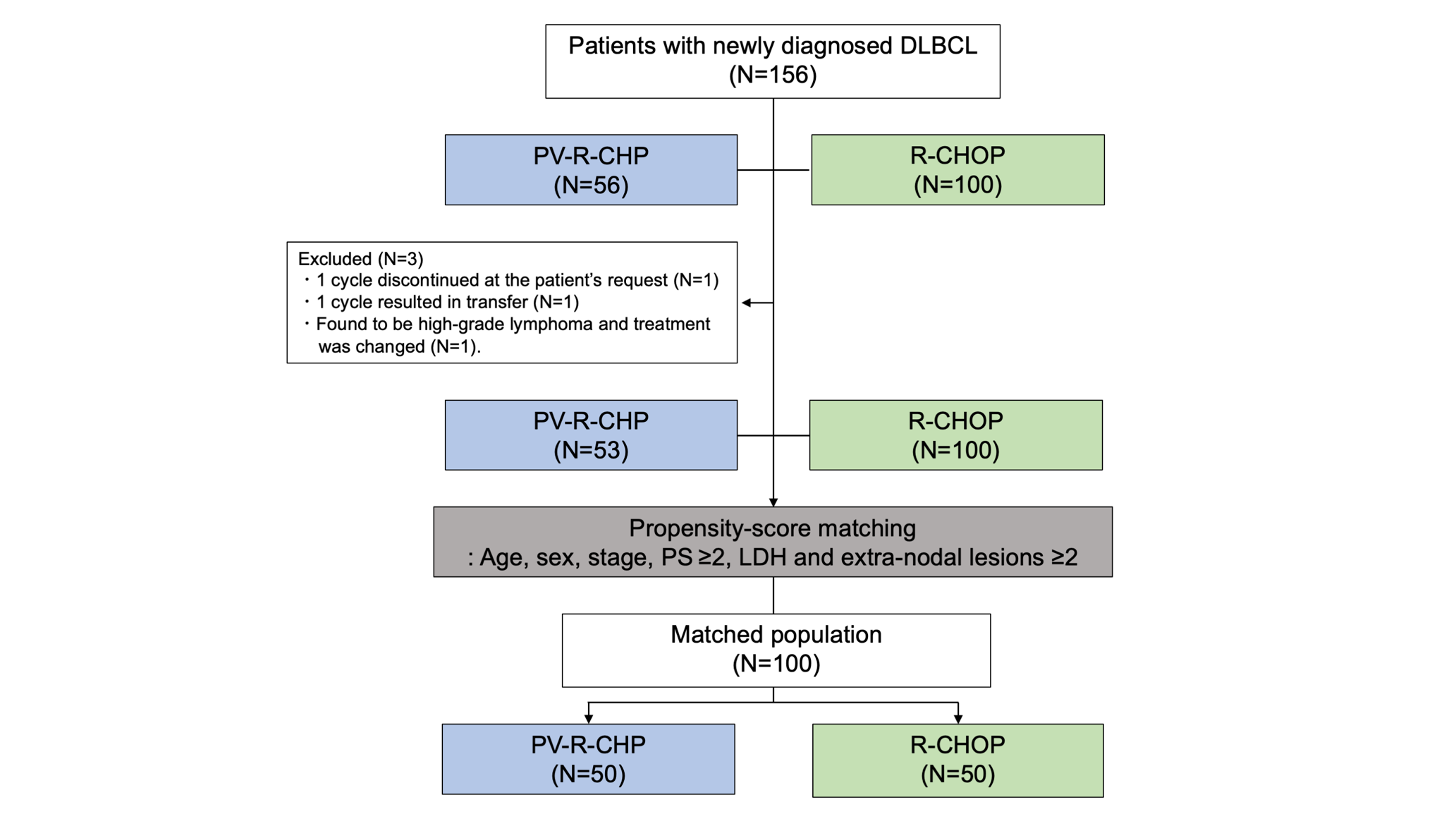
**

**Figure S2 Monthly distribution of PV-R-CHP and R-CHOP-based treatments.**

**
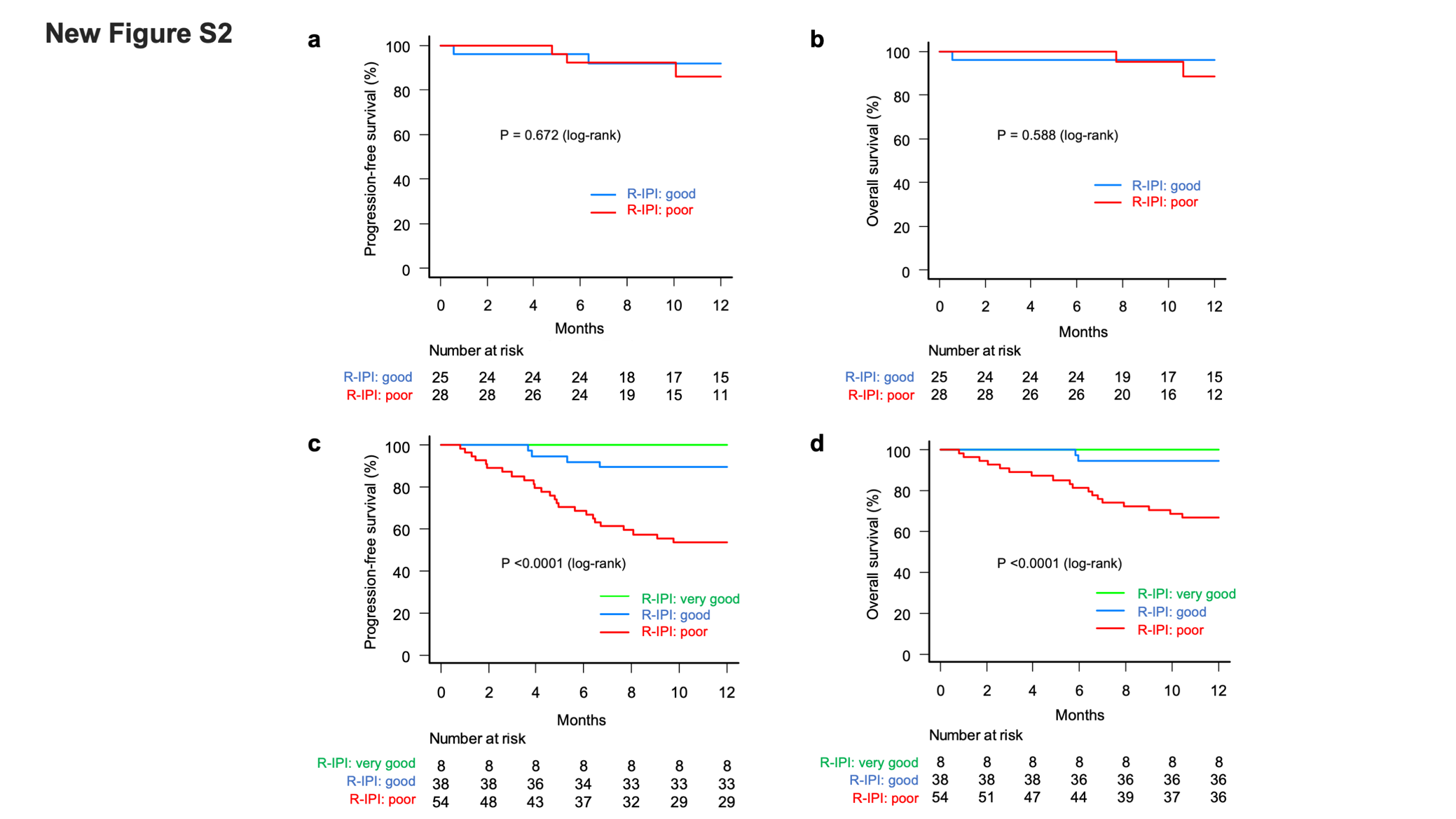
Figure S3 Efficacy outcomes based on the revised International Prognostic Index for patients treated with PV-R-CHP or R-CHOP-based regimens in the overall, unmatched cohort.** Progression-free survival (a) and overall survival (b) in the PV-R-CHP group. Progression-free survival (c) and overall survival (d) in the R-CHOP group


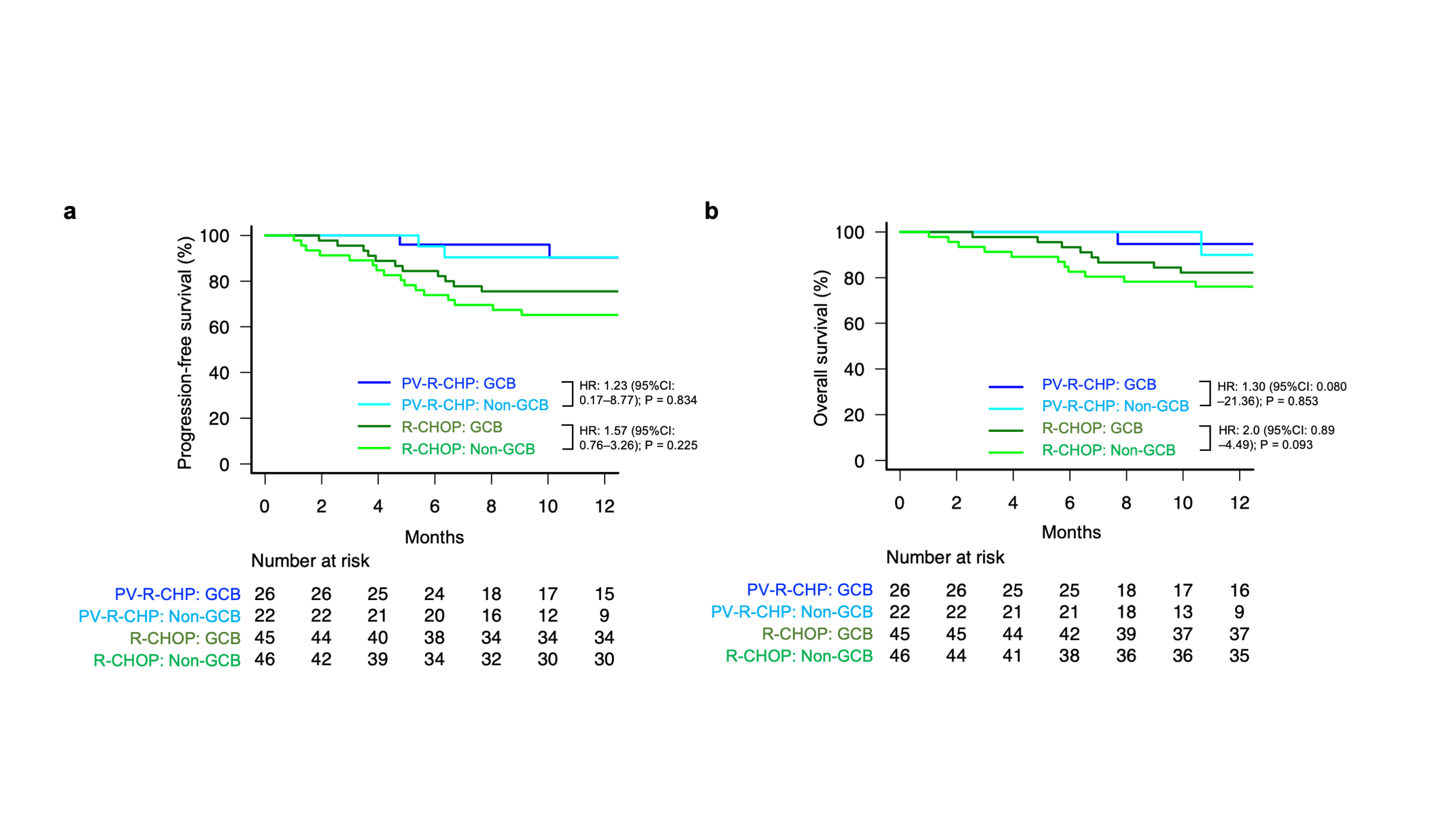
**Figure S4 Survival outcomes for patients treated with PV-R-CHP or R-CHOP-based regimens based on the cell of origin in the overall, unmatched cohort.** Progression-free survival (a) and overall survival (b) in patients with germinal center B-cell-like (GCB) and non-GCB diffuse large B-cell lymphoma.


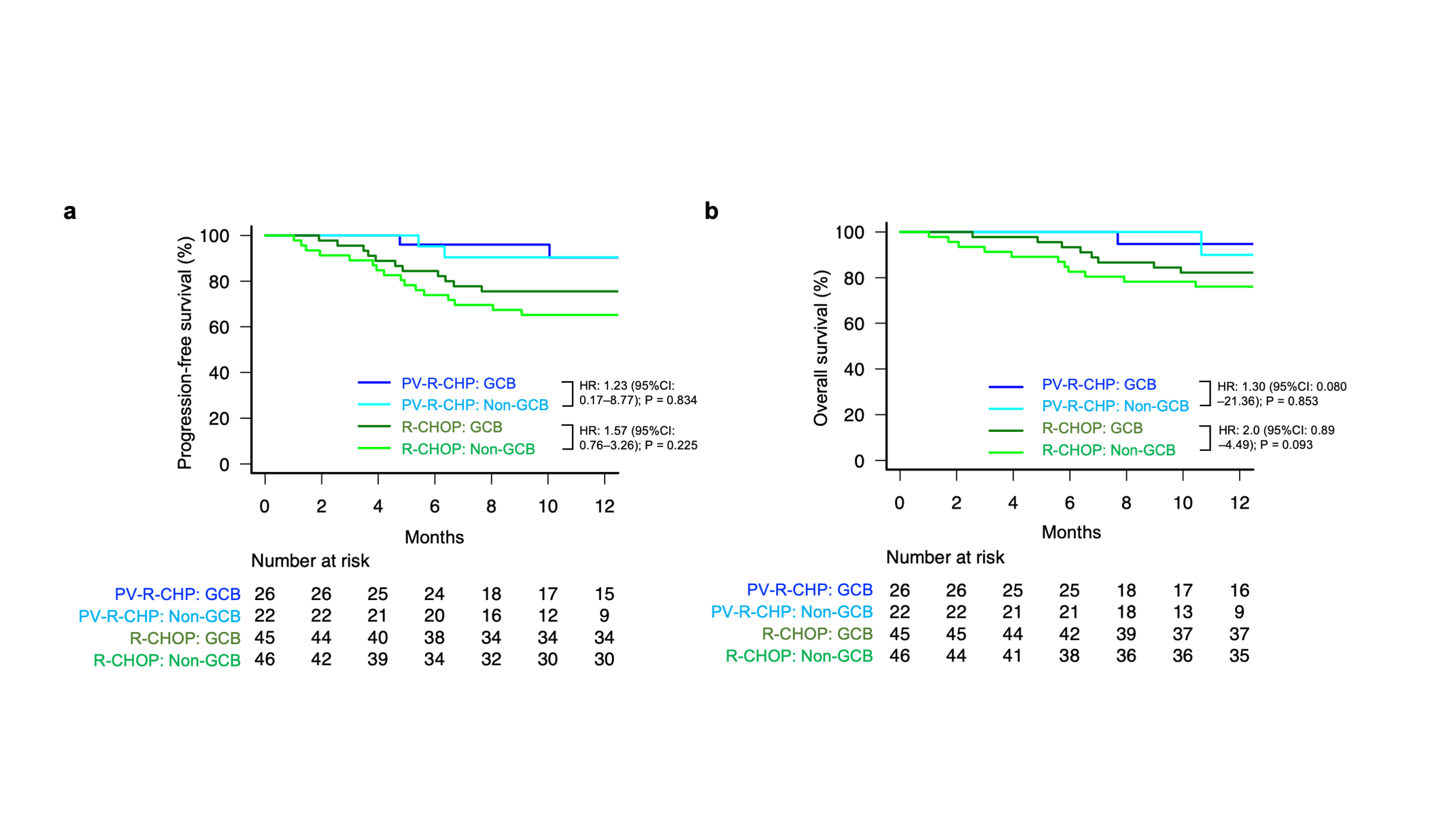


**
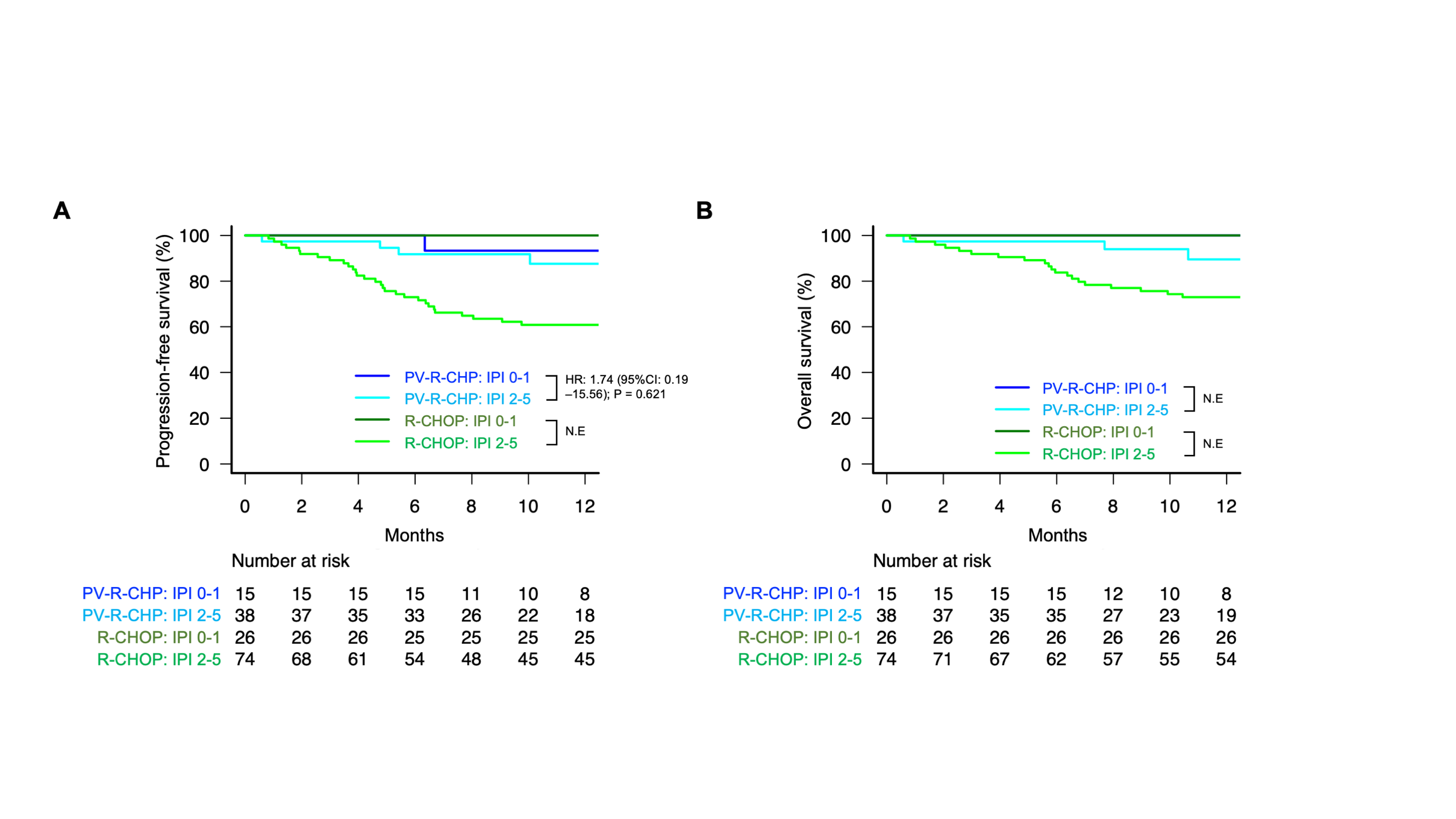

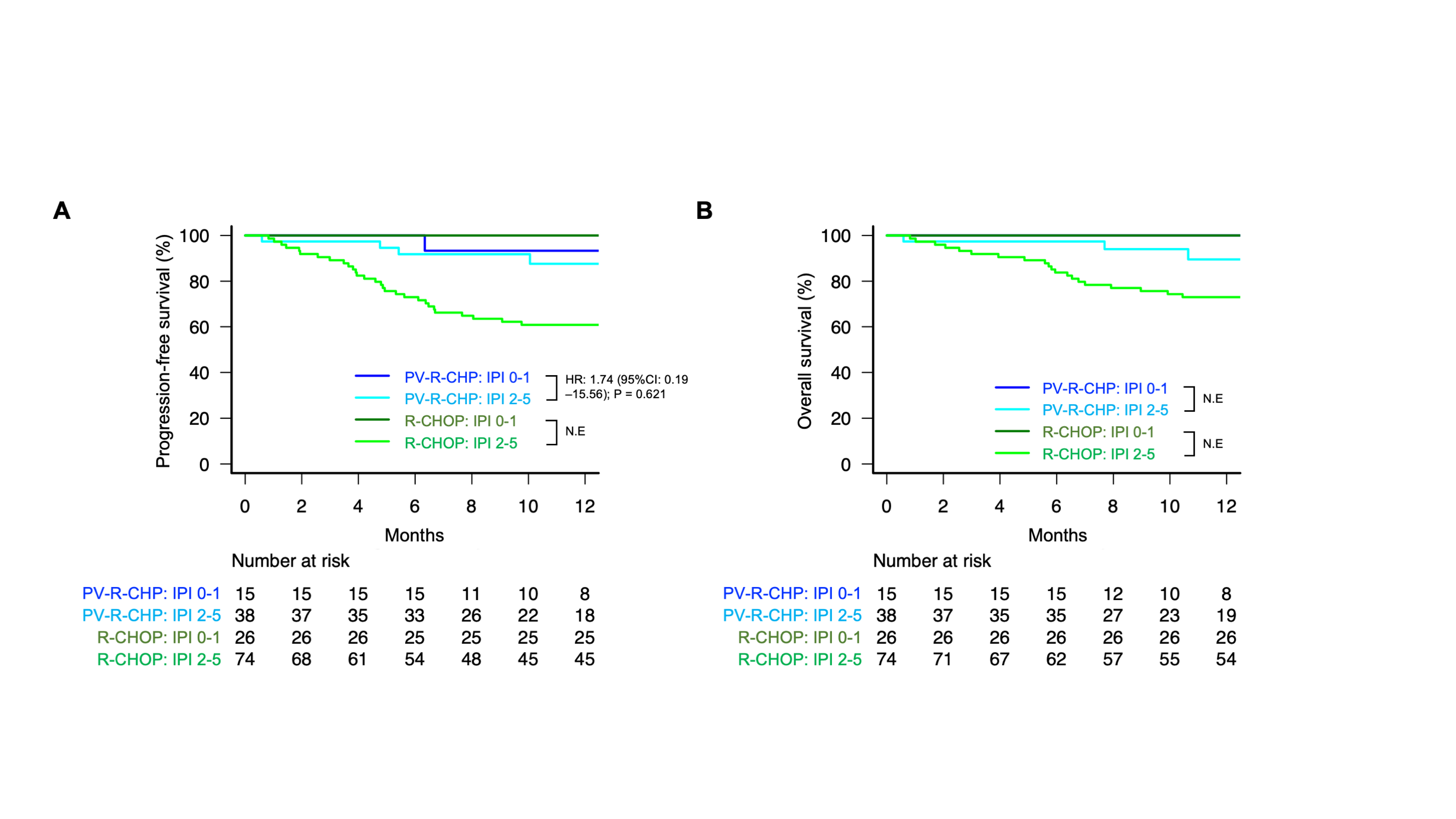
Figure S5 Survival outcomes for patients treated with PV-R-CHP or R-CHOP-based regimens based on the** **International Prognostic Index in the overall, unmatched cohort.** Progression-free survival (A) and overall survival (B).

**b**

**a**

**Figure S6 Survival outcomes for patients treated with PV-R-CHP or R-CHOP-based regimens based on lactate dehydrogenase elevation in the overall, unmatched cohort.** Progression-free survival (A) and overall survival (B).

**
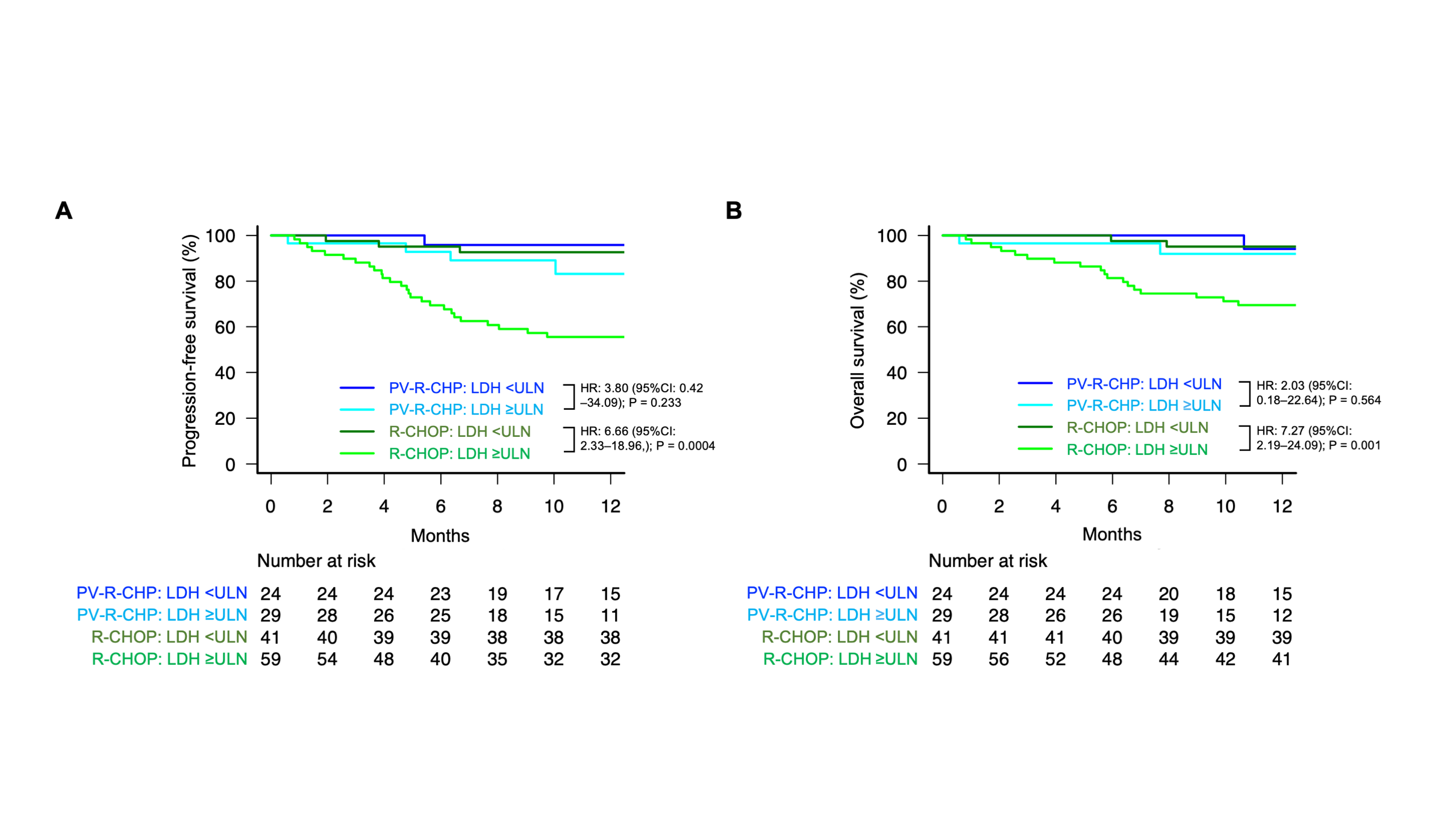
**

**a**

**
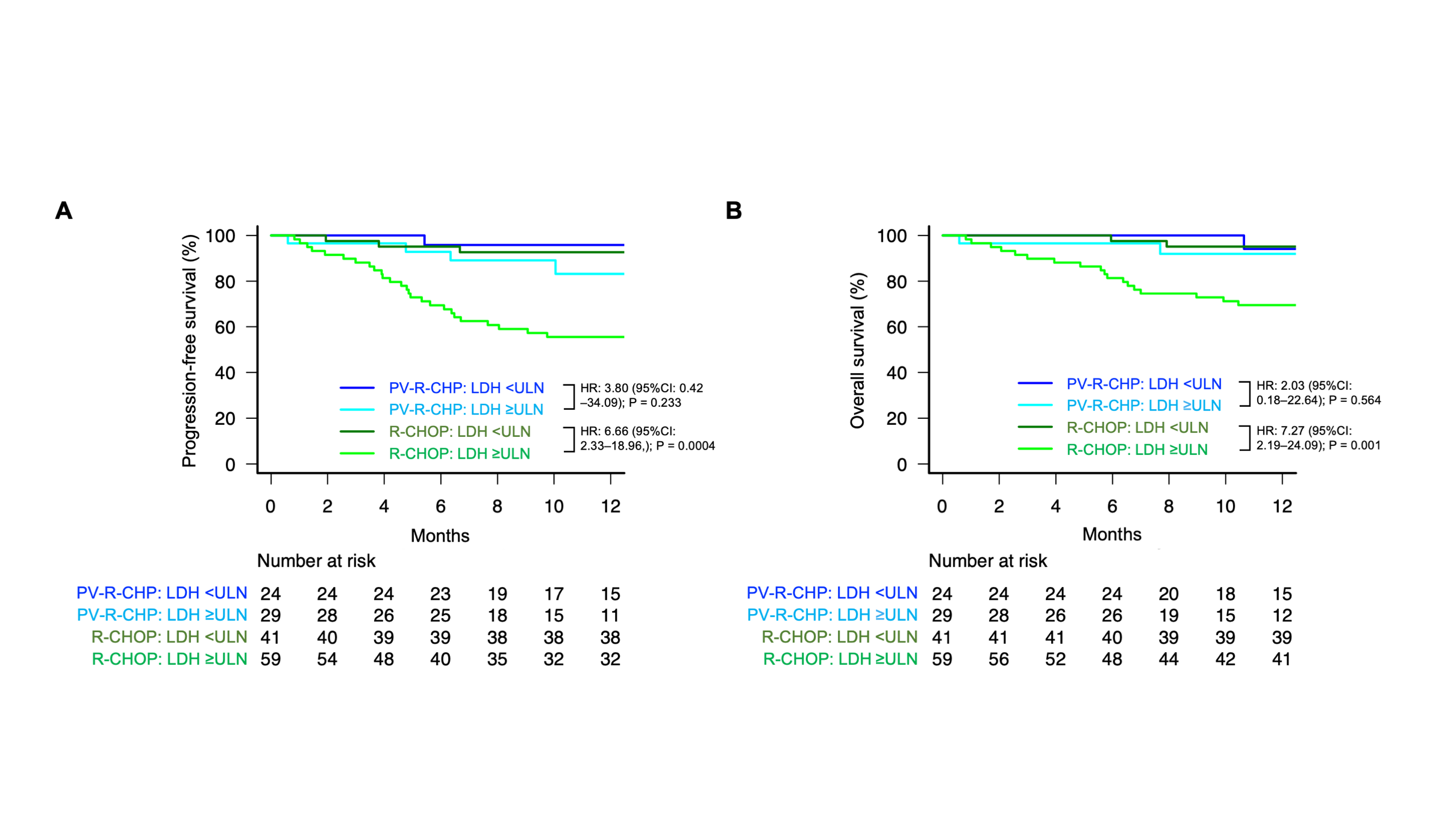
**

**b**

**
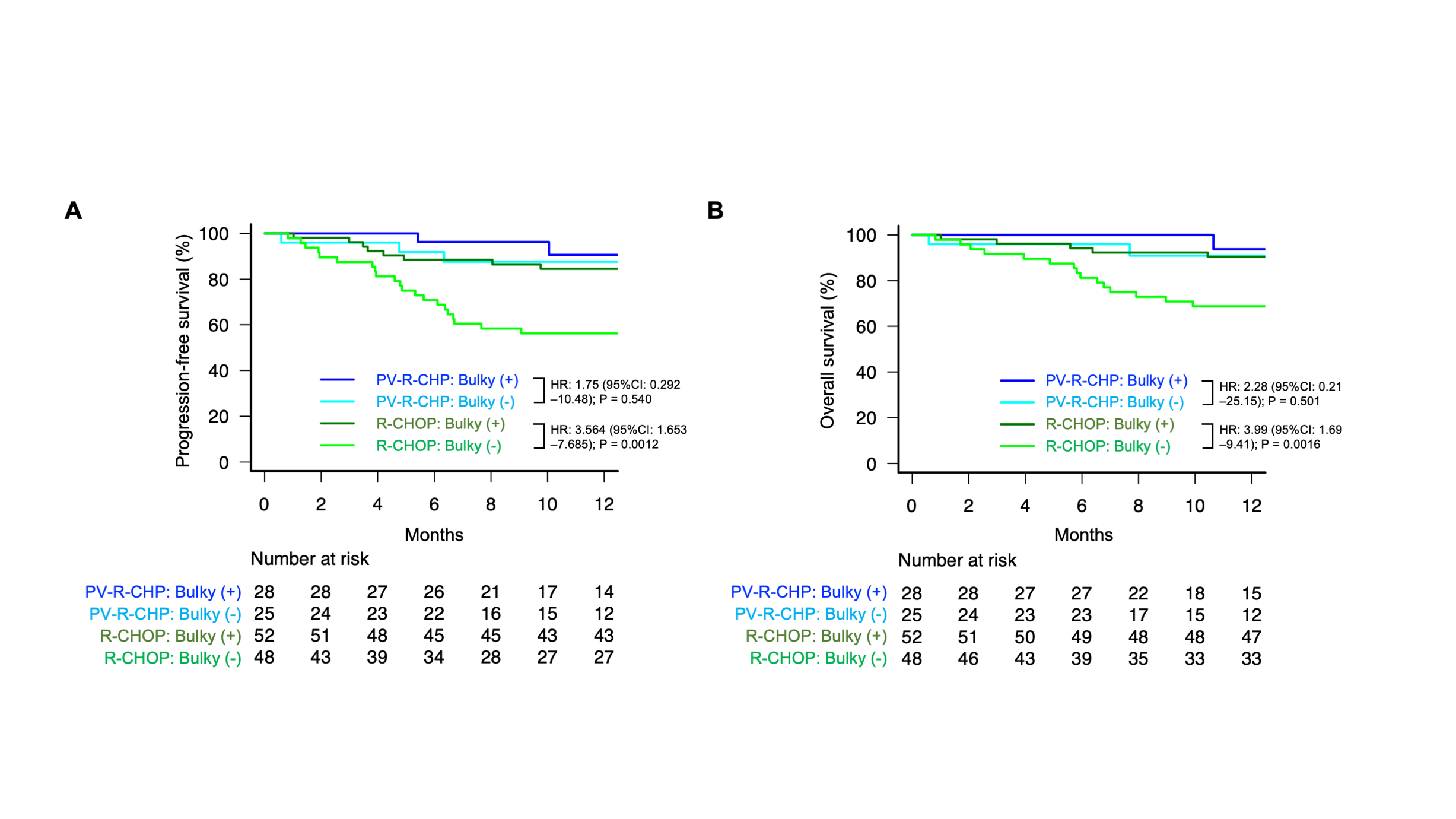
Figure S7 Survival outcomes of patients treated with PV-R-CHP or R-CHOP-based regimens based on the presence of bulky mass lesions in the overall, unmatched cohort.** Progression-free survival (a) and overall survival (b).

**a**

**
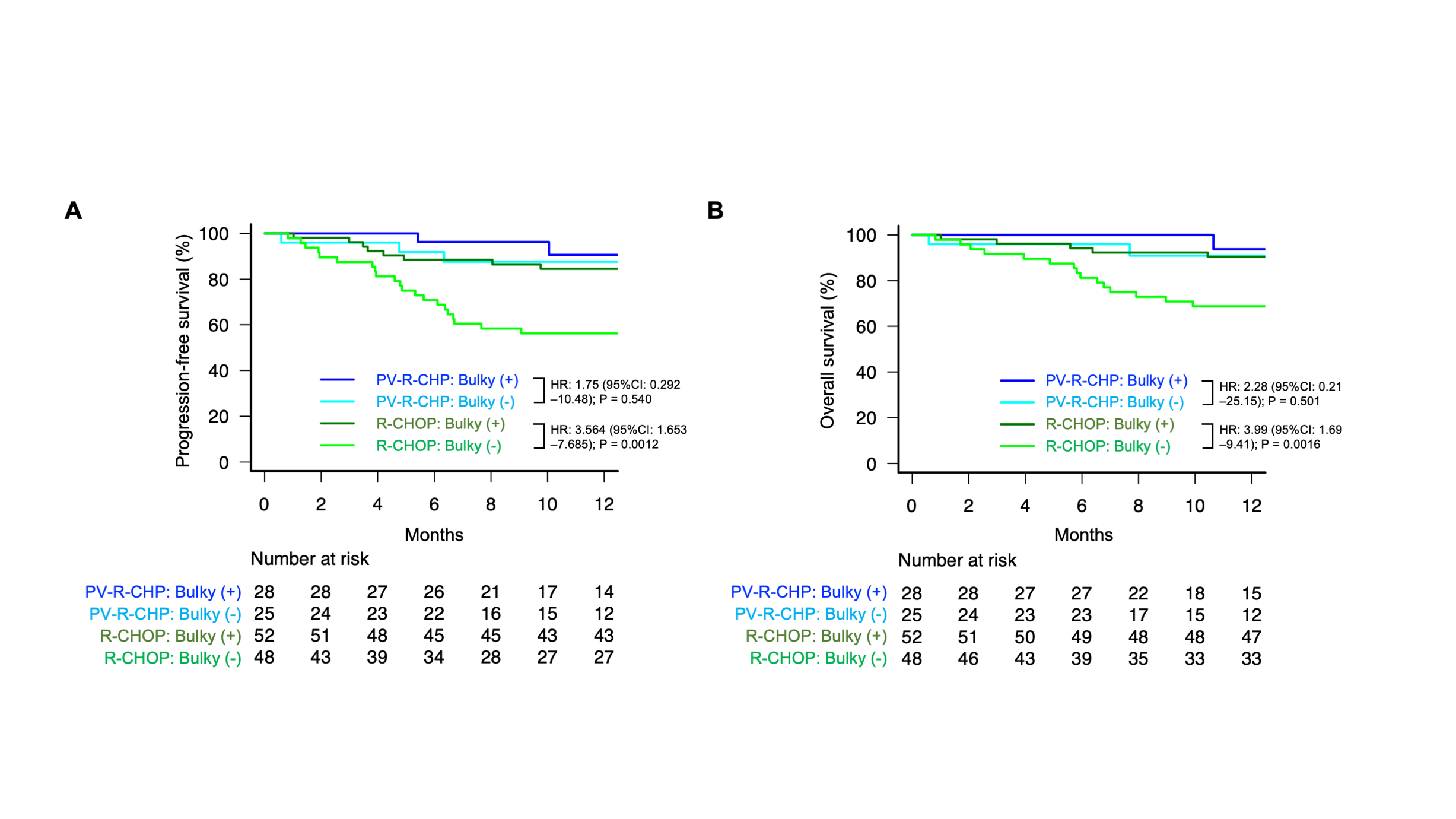
**

**b**

**Figure S8 Butterfly plot showing the proportion of adverse events in propensity score-matched populations.** The R-CHOP group includes patients treated with either R-CHOP or R-THP-COP.


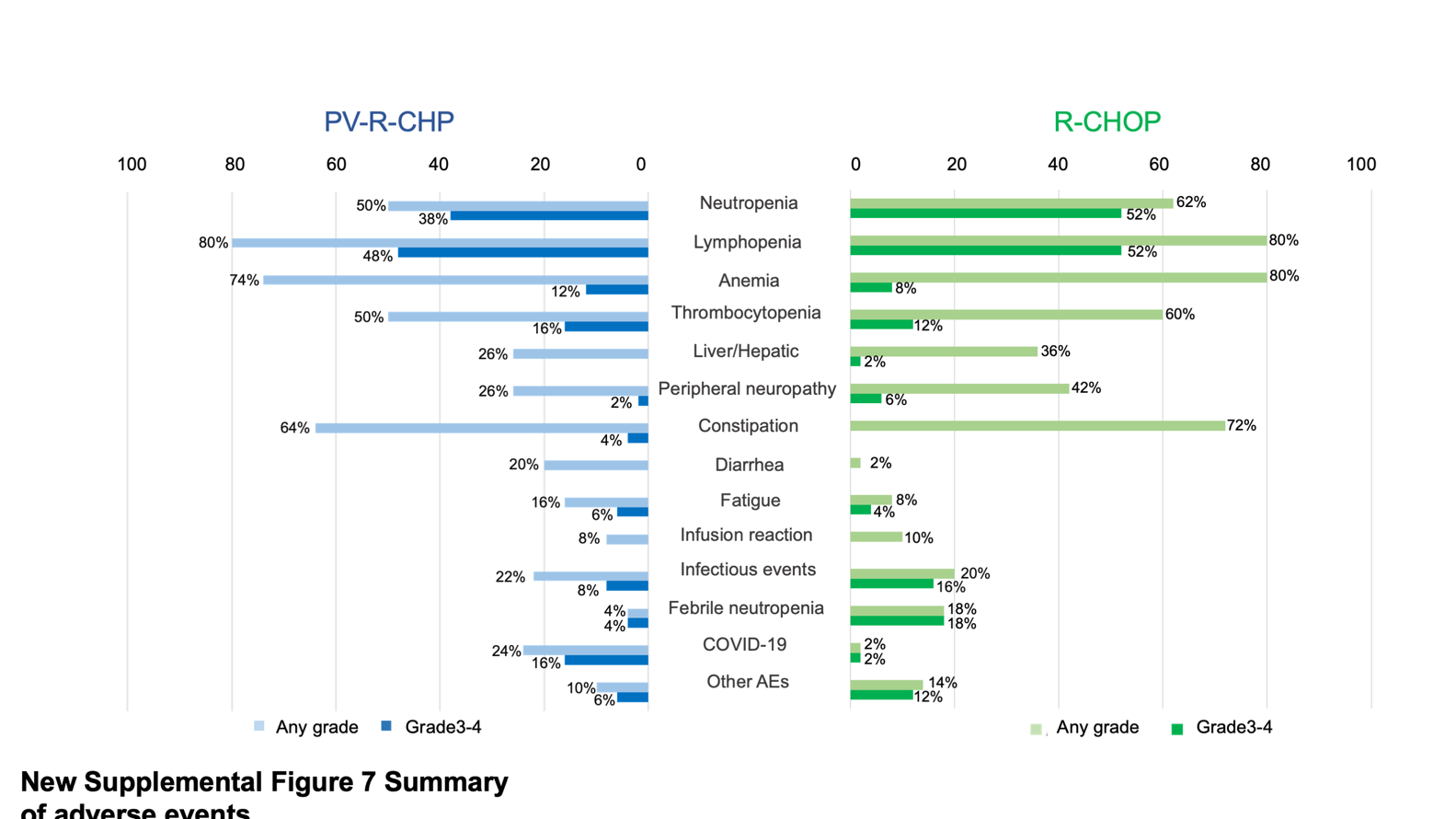

Supplement: Supplementary file 1 — Table S1: Summary of progression‐free and overall survival rates of patients treated with PV‐R‐CHP or R‐CHOP in the overall and propensity score‐matched cohorts. Table S2: Baseline characteristics of propensity score‐matched populations. Table S3: Treatment exposure in propensity‐score matched patients treated with PV‐R‐CHP or R‐CHOP‐based regimens. Figure S1: Overview of patient inclusion and propensity‐score matching. The R‐CHOP group includes patients treated with either R‐CHOP or R‐THP‐COP. Figure S2: Monthly distribution of PV‐R‐CHP and R‐CHOP‐based treatments. Figure S3: Efficacy outcomes based on the revised International Prognostic Index for patients treated with PV‐R‐CHP or R‐CHOP‐based regimens in the overall, unmatched cohort. Progression‐free survival (a) and overall survival (b) in the PV‐R‐CHP group. Progression‐free survival (c) and overall survival (d) in the R‐CHOP group. Figure S4: Survival outcomes for patients treated with PV‐R‐CHP or R‐CHOP‐based regimens based on the cell of origin in the overall, unmatched cohort. Progression‐free survival (a) and overall survival (b) in patients with germinal center B‐cell‐like (GCB) and non‐GCB diffuse large B‐cell lymphoma. Figure S5: Survival outcomes for patients treated with PV‐R‐CHP or R‐CHOP‐based on the International Prognostic Index in the overall, unmatched cohort. Progression‐free survival (a) and overall survival (b). Figure S6: Survival outcomes for patients treated with PV‐R‐CHP or R‐CHOP‐based on lactate dehydrogenase elevation in the overall, unmatched cohort. Progression‐free survival (a) and overall survival (b). Figure S7: Survival outcomes of patients treated with PV‐R‐CHP or R‐CHOP‐based on the presence of a bulky mass lesion in the overall, unmatched cohort. Progression‐free survival (a) and overall survival (b). Figure S8: Butterfly plot showing the proportion of adverse events in propensity score‐matched populations. The R‐CHOP group includes patients treated with either R‐CHOP or R‐TH [file CAM4-15-e71531-s001.docx]
